# In‐Situ HF Forming Agents for Sustainable Manufacturing of Iron‐Based Oxygen Reduction Reaction Electrocatalysis Synthesized Through Sacrificial Support Method

**DOI:** 10.1002/cssc.202401185

**Published:** 2024-11-07

**Authors:** Silvia Mostoni, Lorenzo Mirizzi, Alessandra Frigerio, Giovanni Zuccante, Chiara Ferrara, Mohsin Muhyuddin, Massimiliano D'Arienzo, Sara Fernanda Orsini, Roberto Scotti, Alessio Cosenza, Plamen Atanassov, Carlo Santoro

**Affiliations:** ^1^ Department of Materials Science University of Milano-Bicocca U5 Via Roberto Cozzi 55 20125 Milano Italy; ^2^ Department of Industrial Engineering University of Padova Via Marzolo 9 Padova 35131 Italy; ^3^ Department of Chemical and Biomolecular Engineering University of California Irvine CA 92697 United States

**Keywords:** Oxygen reduction reaction, Sacrificial support method, Avoid HF, Alkaline media electrolyte, Acidic media electrolyte

## Abstract

Fe−N_x_−Cs being suitable to replace scarce and overpriced platinum group metals (PGMs) for cathodic oxygen reduction reaction (ORR) are gaining significant importance in the fuel cell arena. Although the typical sacrificial support method (SSM) ensures the superior electrocatalytic activity of derived Fe−N_x_−C, removing silica hard templates always remains a great challenge due to the hazardous use of highly toxic and not environmentally friendly hydrofluoric acid. Herein, strategic insight was given to modified SSM by exploiting the in‐situ formation of HF, deriving from the decomposition of NH_4_HF_2_ and NaF, to dissolve silica templates, thus avoiding the direct use of HF. First, the suitable molar ratio between the etching agent and the silica was analyzed, revealing that NH_4_HF_2_ efficiently dissolved silica even in a stoichiometric amount, whereas an excess of NaF was required. However, both etching agents exhibited conformal removal of silica while dispersed active moieties within the highly porous architecture of derived electrocatalysts were left behind. Moreover, NH_4_HF_2_‐washed counterparts demonstrated relatively higher performance both in acidic and alkaline media. Notably, with NH_4_HF_2_‐washed Fe−N_x_−C electrocatalyst, a remarkable onset potential of 970 mV (vs RHE) was achieved with nearly tetra‐electronic ORR as the peroxide yield remained less than 10 % in the alkaline medium.

## Graphical abstract

Fe−Nx−Cs are suitable materials to replace platinum group metals (PGMs) for cathodic ORR. Sacrificial support method ensures high electrocatalytic activity of derived Fe−Nx−C. The removal of silica hard templates remains a great challenge due to usage of HF. In this work, the decomposition of NH_4_HF_2_ and NaF is used to dissolve silica templates, thus avoiding the direct use of HF.

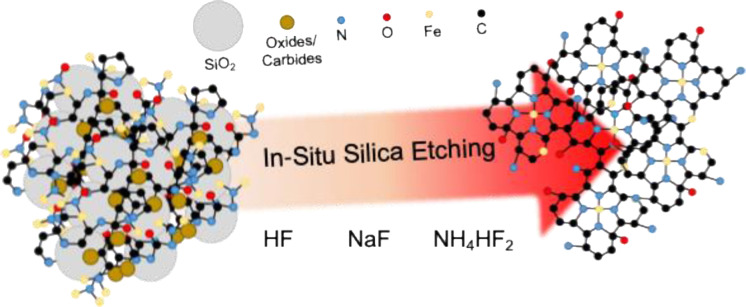

## Introduction

The Hydrogen Economy presents a paramount technology of Fuel Cells (FCs) having the capability to sustainably convert chemical energy into green electrical energy without environmental impacts. The demand for FCs covers a broad spectrum of applications, ranging from domestic to industrial and even automotive sectors. For these scopes, low temperature (T) FCs are preferred to high T FCs because they are more prone to operate with intermittent loading.

The most mature low T FCs are the proton exchange membrane FCs (PEMFCs) that efficiently transform hydrogen into electricity. PEMFCs rely on electrocatalysts based on platinum supported over carbon (Pt/C) on both anode and cathode electrodes.[^1^, ^2^, ^3^, ^4^] On the anode, hydrogen is oxidized in the so‐called hydrogen oxidation reaction (HOR) while on the cathode, oxygen is reduced in the well‐known oxygen reduction reaction (ORR). In PEMFCs, the ORR is sluggish, and therefore, higher Pt/C loading is needed to improve the kinetics and restrict the formation of intermediates while ensuring complete oxygen electro‐reduction.[^5^, ^6^, ^7^] Many improvements have been made in this sense by reducing the Pt/C loading,[^8^, ^9^, ^10^, ^11^, ^12^] improving the electrocatalyst layer interface,[^13^, ^14^] enhancing the electrocatalyst utilization,[^15^, ^16^, ^17^] and so on.[^18^, ^19^, ^20^] It was also tried to substitute Pt/C with first‐row transition metals (TMs) in the form of TM−N_x_−C electrocatalysts.[^21^, ^22^, ^23^] Despite this strategy being the most promising one, many issues are faced by this class of electrocatalysts while operating in acid media as recently summarized in a comprehensive review.^[24]^

In the past 10–15 years, with the development of efficient anion exchange membrane (AEM), the interest and investments in AEMFCs have grown exponentially.[^25^, ^26^, ^27^] In an alkaline environment, TM−N_x_−C electrocatalysts can efficiently substitute Pt/C and therefore much attention has been devoted to this topic. Such electrocatalysts are based on TM−N_x_−C with x=2,3,4 and TM=Fe, Cu, Co, Mn, Ni, etc. integrated into a graphitic‐like structure.[^28^, ^29^, ^30^, ^31^, ^32^, ^33^] These active sites are responsible for a direct 4e^−^ transfer mechanism or for the reduction of the intermediate to the final product.[^34^, ^35^, ^36^] In general, concerning ORR in alkaline media, it was shown that Fe is the most promising electrocatalyst mainly due to its suitable interaction with oxygen.[^37^, ^38^, ^39^] Other active sites can be present on the carbon backbone, such as TM oxides, carbides and/or metallic nanoparticles.[^40^, ^41^] Importantly, also nitrogen moieties play an active role in ORR and in general it can be implied that they are responsible for reducing oxygen through a 2e^−^ transfer mechanism producing the undesired intermediate, H_2_O^−^ + OH^−^.[^40^, ^42^] Pyridinic nitrogen instead has been shown to be active toward the reduction of the intermediate in the final product, OH^−^.^[43]^

Different strategies are presented in the literature for synthesizing these electrocatalysts^[41]^ but, in general, four routes are the most explored and are here briefly presented. The first one uses TM containing azamacrocycles (e. g. phthalocyanine[^44^, ^45^, ^46^, ^47^, ^48^] and porphyrins[^21^, ^49^, ^50^, ^51^]) as precursors that are mixed with a high surface area carbon support and are subjected to pyrolytic processes at different temperatures and different atmospheres.

In another synthetic route, porphyrins or other azamacrocycles can be used successfully as building blocks to create a covalent framework aerogel possessing high surface area and high active site density.[^52^, ^53^, ^54^, ^55^] The covalent framework is subject to supercritical CO_2_ drying and then the aerogel is subject to pyrolysis at a controlled temperature and atmosphere to gain graphitization without losing its intrinsic high porosity.

Another route is based on the utilization of a covalent organic framework (COF) containing TM or metal‐organic framework (MOF). In these cases, COF or MOF are prepared and are subject to pyrolysis processes to enhance graphitization while maintaining the morphological structure and preserving the desired TM−N_x_−C active sites.[^56^, ^57^, ^58^, ^59^, ^60^, ^61^]

The fourth synthetic method considers the mixing of a nitrogen‐rich organic precursor, a metal salt and a templating agent. Soft[^62^, ^63^, ^64^, ^65^] and hard[^66^, ^67^, ^68^] templating are used with the first one being removed during pyrolysis, while the second one after the pyrolytic process. Hard templating is preferred because it helps within the graphitization process and creates a well‐structured porosity.[^69^, ^70^, ^71^] In this context, many nitrogen‐rich organic precursors have been explored along with several metal salt precursors.[^72^, ^73^, ^74^] Hard templating using silica with different sizes and morphologies has been also studied, leading to efficient ORR electrocatalysts.^[75]^ The synthetic route known as the sacrificial support method (SSM) relies on the pyrolytic process in a neutral and/or slightly reducing atmosphere at a temperature above 800 °C, where graphitic carbon formation is enhanced.^[76]^ The removal of the hard templating agent occurs afterward with the support of HF that etch out the silica and create a defined porosity and the pore size distribution depends on the silica size. After silica removal, the ORR electrocatalyst is subjected to a second pyrolysis in a reducing atmosphere.^[77]^ The last synthetic route is also the one that fabricates ORR electrocatalysts at a commercial scale through the company Pajarito Powder.[^69^, ^78^]

While this process is scalable and the ORR electrocatalysts synthesized are among the most efficient ever developed, the biggest bottleneck of the process lies in the utilization of HF, which is difficult to handle and its waste raises remarkable environmental concerns. Recently, the etching of silica was removed from the process by introducing polytetrafluoroethylene (PTFE) within the mixture (N‐rich organic molecules, metal salt, silica templating) that allowed to obtain a defined porosity and efficient ORR^[79]^ and CO_2_ reduction reaction electrocatalyst.^[68]^ This innovative, smart and efficient methodology, prompted us to propose an alternative strategy to remove silica without using HF directly but by exploiting in‐situ forming HF agents such as NH_4_HF_2_ and NaF. In this way, the direct utilization of HF is avoided, reducing the risk of handling a hazardous acid, and leading to an easier and safer synthetic route to fabricate efficient ORR electrocatalysts. The in‐situ formation of HF enables the safer and minimal use of strong acids, while also facilitating the safe disposal of residues. In this framework, we here propose the design and development of a hard templating strategy for the production of Fe−N_x_−C ORR electrocatalysis exploring the effect of two etching agents – NH_4_HF_2_ and NaF/HCl mixture – as in situ forming HF agents and avoiding the direct use of the most hazardous HF and thus opening the way for the sustainable production of ORR electrocatalysts.

## Results and Discussion

### Electrocatalysts Structure and Morphology

This study deals with the development of a more sustainable hard templating removal procedure for the preparation of Fe−N_x_−C ORR electrocatalysts, where Silica‐iron NPs (SFe) were simultaneously used as sacrificial supports to induce the micro‐ and meso‐porosity while homogeneously dispersing iron in the Fe−N_x_−C structure.^[80]^ The structure and composition of silica SFe NPs are in agreement with our previous study,^[80]^ as reported in Figure S1. Compared to the current technology, which entails the use of HF as an etching agent for the removal of SiO_2_‐based templating agents, in this new approach, two different in situ HF forming agents were tested: i) NH_4_HF_2_ and ii) NaF + HCl. This strategy was recently proposed by Gentile *et al*. during the production of MXene^[81]^ but it was never proposed for SSM Fe−N_x_−C family ORR electrocatalysts. Samples were named SFe−Y−X where Y is the etching agent and X the ratio between the etching agent and the silica. SFe−P1 is the electrocatalyst that was subject to the first controlled pyrolysis but did not undergo silica etching.

**Figure 1:**
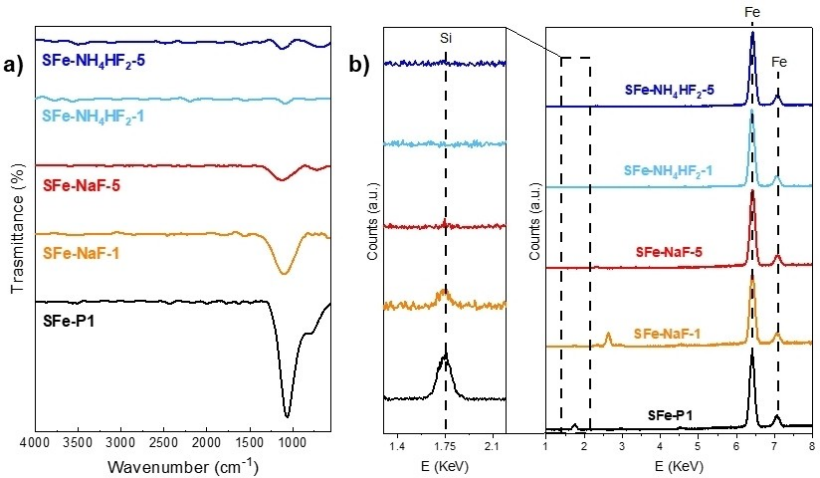
a) ATR‐FTIR and b) XRF spectra obtained for SFe−Y−X materials in comparison to SFe−P1. Magnification of the the Si spectral region in highlighted in b).

The effective removal of SiO_2_ NPs was deeply investigated by using several techniques, including ATR‐FTIR, TGA, XRF, and TEM analyses. ATR‐FTIR spectra of SFe−Y in comparison to SFe−P1 (Figure 1a) first evidence that these salts are efficient in the etching process of SiO_2_: the typical features of SiO_2_, whose main peak is due to the stretching of Si−O−Si bond (ν_Si‐O‐Si_) is located at 1060 cm^−1^ in SFe−P1, disappears after the etching treatment with both NH_4_HF_2_ and NaF at high amount (X=5). However, when smaller amounts of the two agents are used (X=1), only NH_4_HF_2_ maintains the same high performance, while just a reduction in the intensity of the SiO_2_ peak is achieved with NaF.

Similar results are highlighted by XRF analysis (Figure 1b). For all samples the peaks due to Fe are detected at 6.41 KeV and 7.09 KeV,^[82]^ confirming the presence of iron within the electrocatalysts. Besides, the Si signal at 1.74 KeV^[82]^ is observed in SFe−P1, but it becomes imperceptible in the SFe−Y−X series. Only for SFe‐NaF‐1, a tiny peak is still visible (zoom of Figure 1b), confirming that SiO_2_ is only partially etched under these experimental conditions.

**Figure 2:**
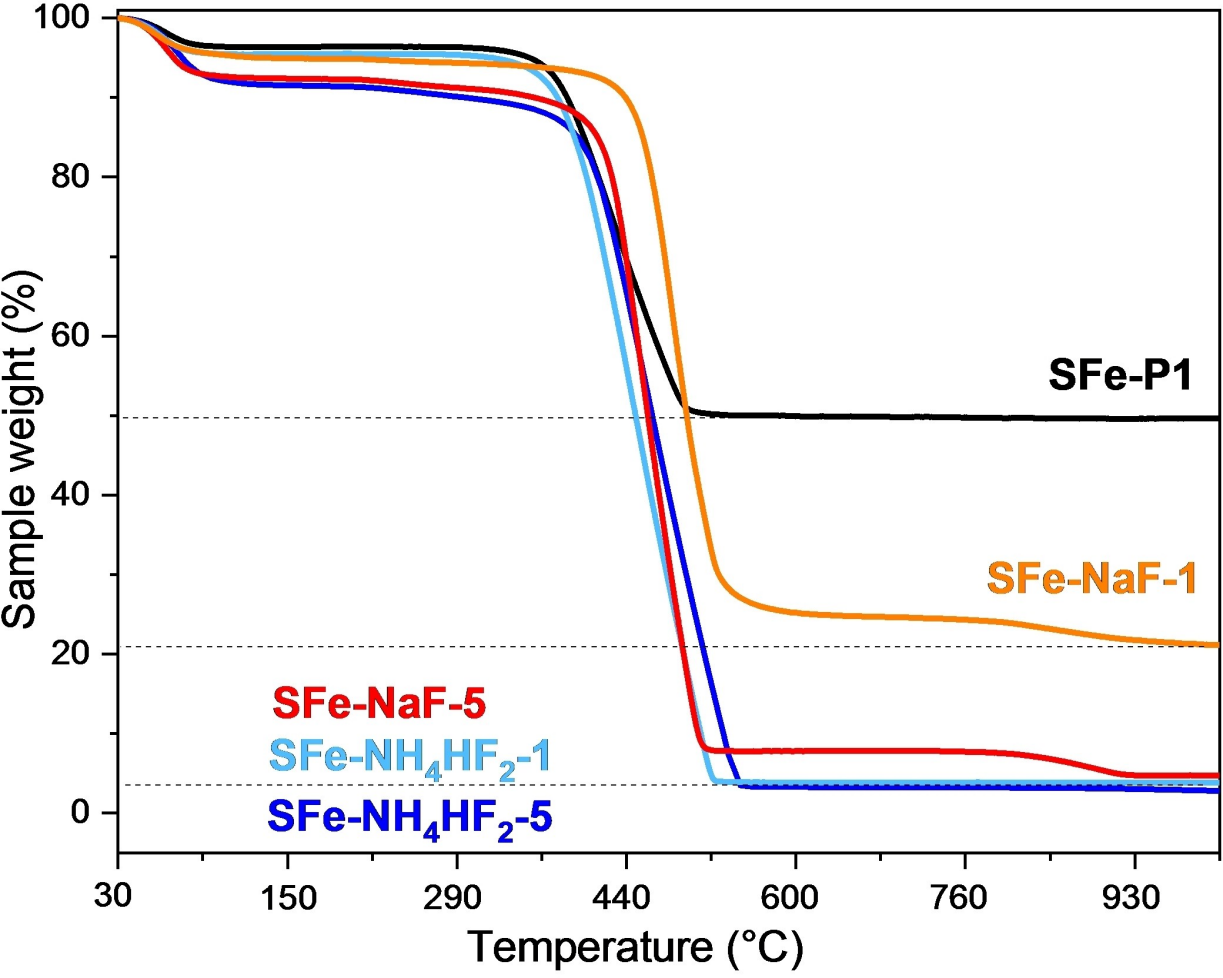
TGA curves of SFe‐Y−X compared to that of SFe−P1.

A quantification of SiO_2_ removal was obtained by TGA analysis performed before and after the HF in situ formation treatment (Figure 2). In SFe−P1 the weight percentage due to silica is calculated by the sample weight (%) measured at 1000 °C, where only the inorganic component is still present, while the organic part has been eliminated through combustion.^[83]^ This corresponds to almost 50 wt % of SFe−P1, which is equal to a mass ratio between the inorganic and organic part of 50 : 50; the reduced mass ratio compared to SFe‐NC (30 : 70) is consistent with the degradation of the nitrogen‐rich organic source (Nicarbazin) due to the first pyrolysis. Interestingly, the use of NH_4_HF_2_ at both molar ratios led to a complete removal of silica, as demonstrated by the tiny residual solid recovered at 1000 °C (<4 wt %). This remaining contribution accounts for the iron amount dispersed in SFe−Y−X materials, which is likely transformed into iron oxide species due to the TGA thermal treatment performed in the air. On the contrary, only the use of an excess of NaF (X=5) led to similar results compared to NH_4_HF_2_, whereas a significant amount of inorganic component (~20 wt %) is still measured at 1000 °C for SFe‐NaF‐1, in agreement with the previous observations in the FTIR and XRF spectra.

These results indicate that both NH_4_HF_2_ and NaF are suitable precursors for the HF in situ formation, necessary for the silica etching, and thus representing potential substitutes to HF in the Fe−N_x_−C preparation. In detail, these salts dissociate in aqueous solution through the following reactions:
(1)

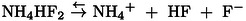



(2)

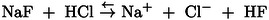




giving rise to in situ HF formation. The so‐formed dilute HF solutions generate the following equilibrium reaction:
(3)

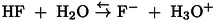




where F^−^ and HF are then able to interact forming:
(4)

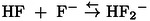




The equilibrium constant at 25 °C of Equation (3) and Equation (4) are reported as K_3_=6.6*10^−4^ and K_4_=3.9, respectively.^[84]^ According to K_3_ and K_4_, in the NH_4_HF_2_ solution, the most predominant fluoride species is represented by HF_2_^−^, which is formed from the reaction of HF and F^−^ ions, directly available from Equation (1). On the contrary, in the NaF solution, the similar concentration of both HF and HF_2_^−^ should be present, as only little amounts of F^−^ are formed through Equation (3) compared to the NH_4_HF_2_ solution, due to the low K_3_. The diversity in the composition of the fluoride‐based solutions could be herein used to explain the higher performances obtained with NH_4_HF_2_ at low content compared to NaF (X=1). Indeed, Judge et al. claimed that the dissolution rate of SiO_2_ in dilute acidic fluoride solutions depends only on the concentration of HF_2_^−^ and HF, whereas no influence of free fluoride was observed.^[85]^ Besides, they claim that the dissolution rate is about four to five times higher with HF_2_^−^ compared to that obtained with HF. Verhaverbeke et al. further validated the previous model by Judge, also suggesting that a dimer of HF, (HF)_2_, is the other most important etching species together with HF_2_^−^,^[84]^ as only the occurrence of 2 or 4 fluorine atoms can initiate the etching process. No other species as (HF)_2_ F^−^, (HF)_3_ F^−^ or (HF)_4_ F‐ are taken into account at low HF concentrations (<1 M).[^86^, ^87^] More recently, new models also included the HF and F^−^ contribution to the silica etching process, still confirming the main role played by the negatively charged HF_2_^−^ species.^[88]^ Thus, the likely higher HF_2_^−^ concentration in the NH_4_HF_2_ solution could favor the kinetic of the etching process of SiO_2_. The discrepancy is almost eliminated by introducing higher amounts of the two etching agents.

In a nutshell, both NH_4_HF_2_ and NaF could represent potential turning points to reduce the overall hazard encountered in the Fe−N_x_−C synthesis due to the direct use of HF and prompt the large‐scale production of Fe−N_x_−C by simplifying the entire process and required facilities. Indeed, it must be stressed that even if the final obtained product if HF, the starting precursors are labeled as corrosive and toxic for ingestion (NH_4_HF_2_) and irritating (NaF), the HF is associated with much more severe risks as it is HF is defined as dangerous, fatal if swallowed, in contact with skin or inhaled and requires much stringent safety procedures when used in laboratory and industrial scale.

Later on, the morphological and structural investigation was centered on SFe‐NH_4_HF_2_–1 and SFe‐NaF‐5 samples, in which the lower amount of both agents was used with the highest performance for silica removal, to unveil their main features.

The structures of the two novel electrocatalysts were studied through XRPD, which show a broad peak at about 25° and a minor one at about 44°, due to the 002‐oriented and 101‐oriented diffraction peaks of graphite, respectively (Figure S2). No other features due to additional phases (i. e. amorphous silica) or impurities are detected, indicating a good homogeneity and purity of the materials at the end of the synthetic process.

**Figure 3:**
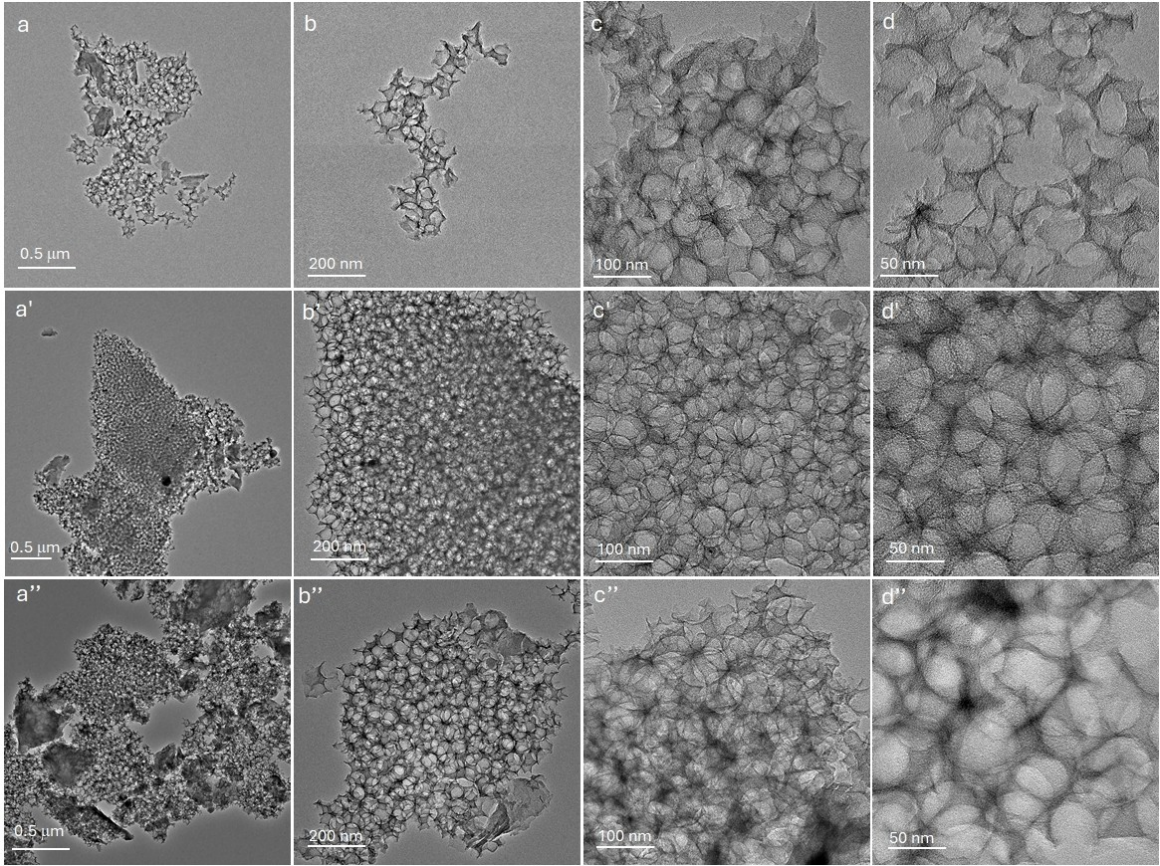
TEM images of SFe‐HF (a‐d), SFe‐NH_4_HF_2_–1 (a’‐d’) and SFe‐NaF‐5 (a’’‐d’’) at different magnifications.

The morphology of SFe‐HF, SFe‐NH_4_HF_2_–1 and SFe‐NaF‐5 samples was investigated through TEM analyses (Figure 3). Images show for all the electrocatalysts the occurrence of homogeneous and continuous graphitic‐based hollow structures made of holes, cavities and discontinuities generated from the removal of silica nanoparticles (Figure 3a‐a’’, b‐b’’). At higher magnifications the superimposition of several macro‐microporous architectures (macropore size estimated from the images of about 65±5 nm) can be appreciated along with the absence of any segregated or crystalline phase (Figure 3c‐c’, d‐d’).

The BET‐specific surface area (SSA_BET_) and pore‐size distribution were determined by nitrogen physisorption. The samples show combined Type I–IV isotherms with narrow H3 hysteresis loop relatable to the presence of micro and mesopores in a macroporous network (Figure S3), in agreement with the hollow architecture observed from TEM images. Both SFe‐NH_4_HF_2_–1 and SFe‐NaF‐5 display large SSA_BET_, namely 495±2 m^2^ g^−1^ and 586±2 m^2^ g^−1^, respectively. In both samples, the contribution of the micropores is relevant (see Table S1) and they represent the majority of the pore population (Figure S4). Conversely, SFe‐HF exhibits a rather lower specific surface area (332±2 m^2^ g^−1^) as well as a minor presence of micropores, as attested by the external surface area calculated by the t‐plot method (Table S1).

### Surface Chemistry

The surface chemistry of the electrocatalyst was thoroughly investigated by means of XPS. All the Fe−N_x_−C samples show a similar surface atomic composition, with a carbon content of about 90.00 at. % and an oxygen content of around 5.50 at. %. The three electrocatalysts show a relatively high nitrogen doping (N at. % >3.50), with the maximum value for the material based on the use of NH_4_HF_2_ (N at. %=4.10±0.20). The detected amount of iron is relatively low (Fe at. % <0.20), but in line with previous studies from the literature.[^89^, ^90^] The results are summarized in Table 1.

**Table 1:** Surface atomic composition in at. % from XPS survey spectra.

At. %	SFe‐HF	SFe‐NH_4_HF_2_–1	SFe‐NaF‐5
C 1 s	90.98±0.10	89.99±0.39	90.91±0.25
N 1 s	3.52±0.14	4.10±0.20	3.76±0.06
O 1 s	5.36±0.04	5.84±0.18	5.24±0.36
Fe 2p_3/2_	0.15±0.01	0.08±0.02	0.10±0.05

**Figure 4:**
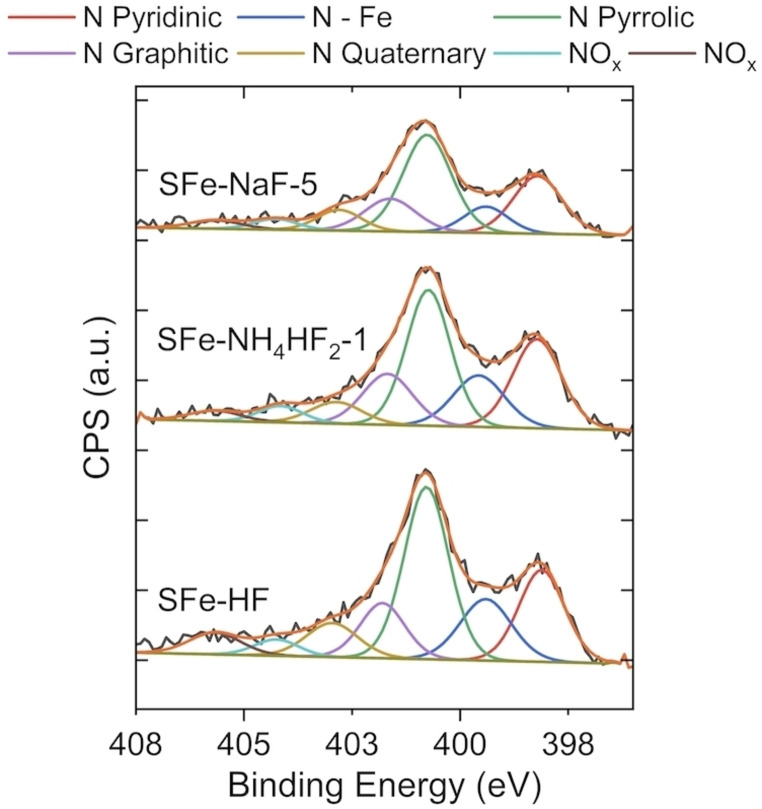
Detailed N 1s spectra of the three electrocatalysts synthesized: SFe‐HF, SFe‐NH_4_HF_2_–1, SFe‐NaF‐5

The high‐resolution N1s and C1s spectra were fitted according to previous works.[^68^, ^89^, ^91^, ^92^, ^93^] From the nitrogen 1 s detailed spectra, shown in Figure 4, several nitrogen moieties were identified: N pyridinic at 398.5 eV, Fe‐nitrogen coordination at 399.4 eV, N pyrrolic at 400.8 eV, N graphitic at 401.8 eV, N quaternary at 403.2 eV and NO_x_ species at 404.4 eV and 405.9 eV. The N 1s relative atomic percentages are presented in Table 2.

**Table 2:** Surface relative composition of nitrogen moieties in relative at. % from XPS N 1s high‐resolution spectra.

Rel. At. %	SFe‐HF	SFe‐NH_4_HF_2_–1	SFe‐NaF‐5
N pyridinic	19.96±0.09	23.66±0.42	23.92±0.82
N−Fe	14.80±0.17	15.36±0.53	10.36±0.85
N pyrrolic	36.24±0.20	34.67±1.14	38.46±0.11
N graphitic	12.05±0.40	12.51±1.76	13.44±0.35
N quaternary	8.48±0.18	6.64±0.39	6.33±1.21
NO_x_	3.50±0.08	4.04±0.04	4.18±0.30
NO_x_	4.98±0.39	3.14±0.16	3.32±0.30

**Figure 5:**
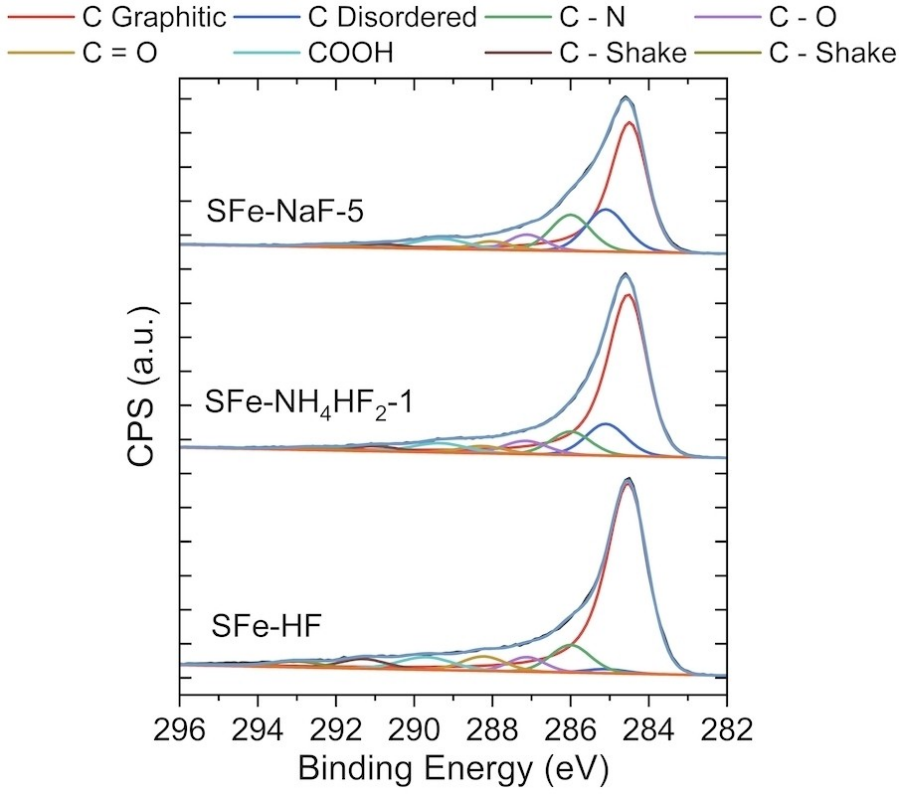
Detailed C 1s spectra of three electrocatalysts synthesized: SFe‐HF, SFe*‐*NH_4_HF_2_–1, SFe‐NaF‐5

The following carbon moieties were identified through the carbon 1 s detailed spectra, presented in Figure 5: graphitic carbon at 284.5 eV, disordered carbon at 285.2 eV, C−N bond at 286.0 eV, C−O at 287.1 eV, C=O at 288.3 eV, COOH at 289.7 eV, C‐F_2_ at 291.3 eV and C‐F_3_ at 293 eV. The C 1s relative atomic percentages are summarized in Table 3.

**Table 3:** Surface relative composition of carbon moieties in relative at. % from XPS C 1s high‐resolution spectra.

Rel. At. %	HF	NH_4_HF_2_	NaF
C graphitic	65.51±0.17	62.59±1.99	52.68±4.53
C disordered	1.94±0.44	10.18±2.52	14.66±3.36
C−N	9.70±0.23	9.12±0.23	13.81±1.65
C−O	5.40±0.61	5.86±0.14	5.92±0.27
C=O	5.26±0.37	3.72±0.58	3.80±0.39
COOH	5.97±0.09	4.81±0.17	5.51±0.32
C−F_2_	3.90±0.11	2.51±0.13	2.31±0.43
C−F_3_	2.35±0.08	1.23±0.12	1.31±0.25

The sample based on the use of HF (SFe‐HF) shows the highest amount of iron and a relatively high content of N−Fe bond, reasonably resulting to be the material with the highest Fe−N_x_ active site density based on the XPS analysis. Instead, following the previous observation, the NaF‐based electrocatalyst is the one with the lowest Fe‐N_x_ active site density. ICP also showed a similar trend of iron content present in the samples as can be seen in Table S2. SFe‐HF had maximum Fe present (2.49 %) followed by SFe*‐*NH_4_HF_2_–1 (1.93 %) whereas the sample NaF‐treated electrocatalyst had the lowest iron content (1.78 %).

## Electrochemical Results

### Oxygen Reduction Reaction in Acid Media

**Figure 6:**
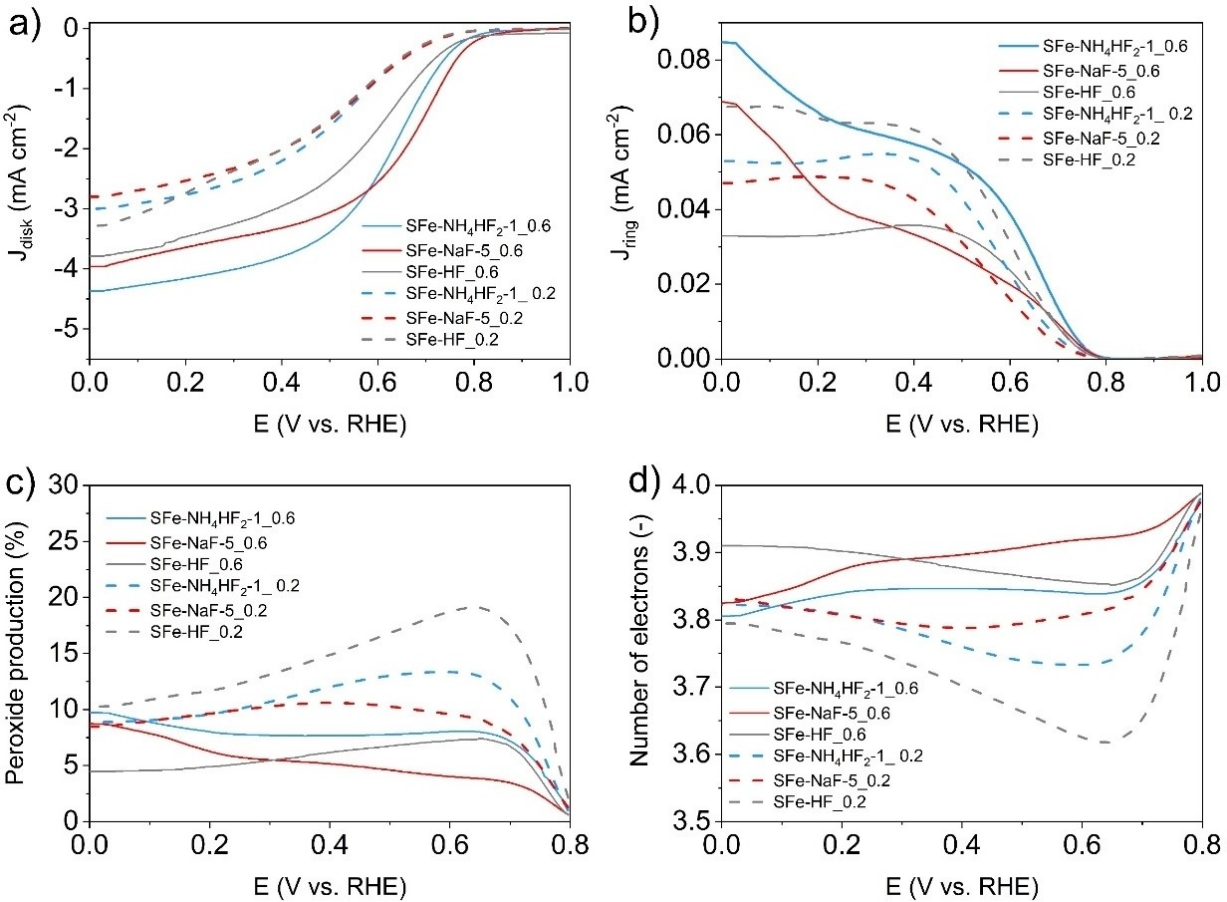
Disk Current density (a), Ring Current density (b), Peroxide produced (c) and Electrons transferred (d) for electrocatalyst etched using HF (grey lines), NaF (red lines) and NH_4_HF_2_ (light blue lines). Two loadings were explored, 0.2 mg cm^−2^ (dot line) and 0.6 mg cm^−2^ (continuous line). Tests were done in 0.5 M H_2_SO_4_ with a rotating speed of 1600 rpm.

ORR electrocatalytic activity of the three electrocatalysts (SFe‐HF, SFe‐NH_4_HF_2_–1 and SFe‐NaF‐5) was evaluated in acidic media, with the electrolyte being 0.5 M H_2_SO_4_. The linear sweep voltammetry was run from 1 V vs RHE to 0 V vs RHE at a rotating rate of 1600 rpm. Two electrocatalyst loadings were investigated, being 0.2 mg cm^−2^ and 0.6 mg cm^−2^. Disk currents are presented in Figure 6a. Interestingly, the increase in electrocatalyst loading led to an increase in both potential onset and half‐wave potential (Figure 6a). At lower electrocatalyst loading, SFe‐NaF‐5_0.2 had an E_on_ of 0.75 V (vs RHE) and an E_1/2_ of 0.55 V (vs RHE). Identical E_on_ but higher E_1/2_ (0.57 V vs RHE) was recorded for both SFe‐NH_4_HF_2_–1_0.2 and Sfe‐HF_0.2. The limiting current (J_lim_) for tests at 0.2 mg cm^−2^ electrocatalyst loading was in the range of 2.8 A cm^−2^ and 3.3 A cm^−2^. At electrocatalyst loading of 0.6 mg cm^−2^, the E_on_ varied between 0.81 V vs RHE (SFe‐NH_4_HF_2_–1_0.6) and 0.85 V vs RHE (SFe‐HF_0.6). The E_1/2_ varied between 0.63 V vs RHE (SFe‐HF_0.6) to 0.65 V vs RHE (SFe‐NH_4_HF_2_–1_0.6) and 0.71 V vs RHE (SFe‐NaF‐5_0.6). The higher J_lim_ was recorded with SFe‐NH_4_HF_2_–1_0.6 and it was 4.4 mA cm^−2^. Ring current density was recorded and presented in Figure 6b. From the ring current density, it was possible to determine the peroxide produced (Figure 6c) that varied between 4 % and 15 % at potentials below 0.4 V vs RHE. The increase in electrocatalyst loading led to a decrease in peroxide produced indicating that the peroxide is consumed within the thicker electrocatalyst layer. The number of electrons transferred was always above 3.7 (Figure 6d). This indicates that the ORR occurs mainly over a direct 4‐electron transfer.

### Oxygen Reduction Reaction in Alkaline Media

**Figure 7:**
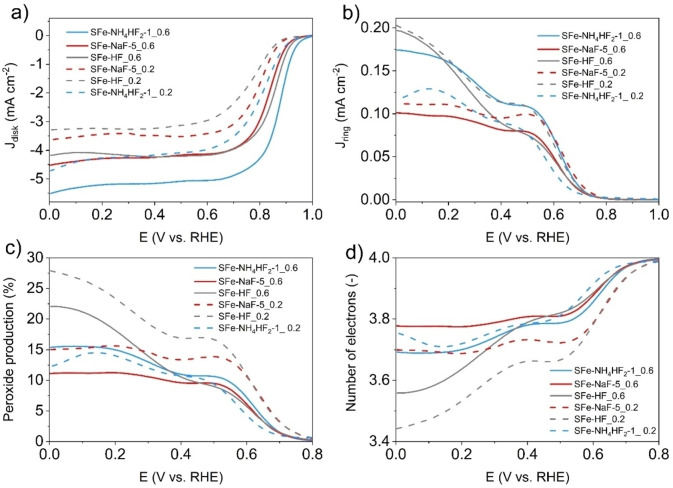
Disk Current density (a), Ring Current density (b), Peroxide produced (c) and Electrons transferred (d) for electrocatalyst etched using HF (grey lines), NaF (red lines) and NH_4_HF_2_ (light blue lines). Two loadings were explored, 0.2 mg cm^−2^ (dot line) and 0.6 mg cm^−2^ (continuous line). Tests were done in 0.1 M KOH with a rotating speed of 1600 rpm.

The electrocatalytic activity of Fe−N_x_−C electrocatalysts in alkaline media is higher compared to the one in acid media[^75^, ^94^] and this was shown also in this case (Figure 7). Disk current is presented in Figure 7a, ring current is shown in Figure 7b, peroxide production is displayed in Figure 7c and transferred electrons are presented in Figure 7d. E_on_ and E_1/2_ increased with the electrocatalyst loading.

SFe‐NH_4_HF_2_–1 had higher results compared to the other electrocatalysts in both loadings. Particularly, SFe‐NH_4_HF_2_–1_0.2 showed an E_on_ of 0.94 V vs RHE and E_1/2_ of 0.85 V vs RHE. At 0.6 mg cm^−2^, SFe‐NH_4_HF_2_–1_0.6 had an E_on_ of 0.97 V vs RHE and E_1/2_ of 0.88 V vs RHE. Interestingly, at the loading of 0.2 mg cm^−2^, SFe‐NaF‐5_0.2 outperformed SFe‐HF_0.2, but at higher loading, SFe‐HF_0.6 had slightly higher electrocatalytic activity compared to SFe‐NaF‐1_0.6 (Figure 7a). A lower peroxide generation was recorded by SFe‐NaF‐5 followed by SFe‐NH_4_HF_2_–1 and SFe‐HF, for which the highest peroxide yield was detected (Figure 7c). The higher number of electrons transferred was achieved by SFe‐NaF‐5 and SFe‐NH_4_HF_2_–1 samples (Figure 7d). Higher electrocatalyst loading led to a higher number of transferred electrons as lower peroxide was detected due to its reduction within the electrocatalyst layer. In general, despite the loading, in alkaline media, the removal of silica through NH_4_HF_2_ results in electrocatalysts with superior features compared to those obtained after etching with HF.

While coming to the point, it is important to highlight that both reagents i. e. NaF and NH_4_HF_2_ proved to be very effective in dissolving the silica templates while maintaining the architectural features and corresponding electrocatalytic activity similar or even superior to that treated by HF. Compared to NaF, five times less NH_4_HF_2_ was consumed to remove silica. Moreover, the NH_4_HF_2_‐treated sample kinetically outperformed the other counterpart (NaF‐treated) by realizing higher ORR E_on_ and E_1/2_ in both electrolytic media i. e. acidic and alkaline. However, the activity was clearly enhanced under alkaline conditions which is aligned with the ultimate goal of AEMFC applications. The peak ORR kinetic activity i. e. E_on_ and E_1/2_ can be linked with the relative higher proportion of Fe−N_x_ in the NH_4_HF_2_‐treated sample as revealed by XPS. Fe−Nx are known as the primary active sites for the ORR.[^43^, ^95^, ^96^] Also ICP confirmed the higher iron content in the NH_4_HF_2_‐treated sample than the NaF‐treated sample. Moreover, TEM illustrated the well‐defined mesoporous graphitic structure without large‐scale coalescence of Fe‐type species into metallic nanoparticles, especially for the SFe‐NH_4_HF_2_–1 sample. Additionally, SFe‐NH_4_HF_2_–1 exhibited a BET surface area that was considerably higher than that of the HF‐treated sample. On the other hand, a slightly higher content of graphitic nitrogen could be a cause of delaying the E_on_ and E_1/2_ of the NaF‐etched sample.^[97]^ However, a slightly higher surface area of the NaF‐treated sample might ensure enhanced accessibility to the active moieties and could be the reason behind the marginally lower peroxide yield. Nevertheless, it should be noted that five times more NaF was required to effectively remove silica templates which could be a limitation. In any case, the performance of the NH_4_HF_2_‐treated sample outperformed the other counterparts in terms of activity while following a nearly tetra‐electronic pathway and keeping the peroxide yield below ~10 % and ~15 % in acidic and alkaline media, respectively.

### Comparisons with Existing Literature

The results presented are in line and sometimes outperform the best PGM‐free electrocatalysts shown in literature for oxygen reduction reactions. In Table 4, a few examples of electrocatalysts tested using SSM and removal of the hard templating using HF are reported. Commercially available Fe−N_x_−C electrocatalysts are also compared.

**Table 4:** Comparison with recent studies involving the etching of silica templates to form ORR electrocatalysts.

Electrocatalysts	Etching Agent	E_on_ (V vs RHE)	E_1/2_ (V vs RHE)	Peroxide (%)	Electrons Transfer	Electrolyte	Catalyst Loading (mg cm^−2^)	Ref.
Fe_AD_−N−C^AF^	Teflon powder	0.9	0.81	1.10	3.9	0.1 M KOH	0.6	[89]
FeNSC	HF solution	0.97	0.87	‐	3.98	0.1 M KOH	0.67^*^	[98]
HPC−Fe/N‐700	NaOH	0.92	0.84	5.4	3.89	0.1 M KOH	0.24	[99]
Ordered mesoporous carbon	NaOH	0.83	0.75	25	2.4	0.1 M KOH	0.2	[100]
Co‐TpBpy‐800	6 M KOH	0.91	0.83	‐	ca. 3.9	0.1 M KOH	0.25	[101]
Si−Fe−N/C	HF solution	‐	0.83	3.35	ca. 3.9	0.1 M KOH	ca. 0.6	[102]
NFe‐PG	NaOH solution	1.00	0.84	‐	3.87–3.99	0.1 M KOH	0.04	[103]
Fe(0)/FeN_x_‐NC‐7	HF solution	0.95	0.86	≤12.3	3.72–3.9	0.1 M KOH	ca. 0.3	[104]
FeNC_PME	HF solution	~ 0.83	0.75	ca. ≥30	3–3.5	0.1 M KOH	0.6	[105]
SEFe_M_P1AP2	HF solution	0.96	0.88	ca. ≤14.5	ca. 3.7–3.9	0.1 M KOH	0.6	[80]
(FeCo)HPNC@NaCl	3 M KOH	‐	0.81	‐	3.92	0.1 M HClO_4_	0.51	[106]
NSMC 0.4	2 M NaOH	0.78	0.68	81	1.8	0.1 M KOH	0.204	[107]
2 %Fe‐ZIF@NaCl	HCl	0.96	0.83	‐	ca. 4	0.1 M HClO_4_	0.8	[108]
FeCo‐OMPC	10 % HF	1.00	0.85		3.9	0.1 M HClO_4_	0.6	[109]
SFe‐NH_4_HF_2_–1_0.6	NH_4_HF_2_	0.97	0.88	≤15	≥3.81	0.1 M KOH	0.6	This work
SFe‐NH_4_HF_2_–1_0.6	NH_4_HF_2_	0.81	0.65	<10	>3.8	0.5 M H_2_SO_4_	0.6	This work

*Estimated Electrocatalyst Loading.

### Outlook Towards Sustainable Large‐Scale ORR Electrocatalysts

In this work, Fe−N_x_−C‐type electrocatalysts were synthesized through the sacrificial support method (SSM). The main variation was the removal of the hard templating with different agents. While HF is generally used for this procedure, the utilization of other agents that can produce HF in situ is novel and not yet explored. The usage of HF is considered a critical bottleneck for the SSM synthetic procedure because HF is extremely difficult to handle, it is extremely volatile and if not handled properly it can cause extremely severe health injuries and environmental issues. Therefore, alternative solutions that are also more sustainable have to be considered. In this work, the etching was conducted also with NaF and NH_4_HF_2_. While the etching with NaF was done by mixing NaF with HCl, a water solution containing NH_4_HF_2_ was used limiting the handle of dangerous agents. Even HBF_4_ was used in this work (data not shown), however the removal of silica was extremely poor. In this study, the etching using an aqueous solution containing NH_4_HF_2_ was more effective than using HF and this was proven by higher Nx−Fe relative percentage (primary active sites) and by the higher percentage of N‐pyridinic (secondary active sites) compared to the other two electrocatalysts tested. Moreover, the NH_4_HF_2_ etched electrocatalyst showed similar performance compared to HF in acid media but superior electrocatalytic activity in alkaline media.

## Experimental Section

### Materials

For the synthesis of silica templates: tetraethylorthosilicate (TEOS), ammonium hydroxide (NH_4_OH 25 %) and iron sulphate eptahydrate Fe_2_(SO_4_)_3_ ⋅ 7H_2_O were purchased from Merck Life Science (Germany). N‐aminoethyl‐aminopropyltriethoxysilane (EDTMS) and Ethanol (EtOH, 99.8 %) were obtained from abcr Gute Chemie (Germany) and Exacta Optech LabCenter (Italy), respectively. For the electrocatalyst preparation, nicarbazin (NC), ammonium hydrogen difluoride (NH_4_HF_2_, 95 %), sodium fluoride (NaF, ≥99 %) and hydrochloric acid (HCl, 37 % v/v) were acquired from Merck Life Science (Germany). Milli‐Q water was produced by a Milli‐Q Essential apparatus.

### Electrocatalyst Synthesis

The electrocatalysts were prepared by means of the hard template approach, using functionalized SiO_2_ NPs as sacrificial supports for the dispersion of iron active centers and to induce the micro‐ and meso‐porosity into the electrocatalyst structure.^[80]^ Briefly, SiO_2_ NPs (average diameter 70±5 nm) were prepared through a Stöber method[^110^, ^111^] and surface functionalized by the hydrolysis and condensation of EDTMS with the surface ‐OH groups of SiO_2_ NPs (molar ratio between silane: OH groups of SiO_2_ equal to 1 : 2). Then, the terminal amino groups of EDTMS were exploited to disperse single iron sites (using Fe_2_(SO_4_)_3_ ⋅ 7H_2_O as iron precursor) onto the SiO_2_ surface (Fe:EDTMS molar ratio equal to 1.5 : 1). The final material was labeled as SFe and used to synthesize Fe−N_x_−C electrocatalysts.

SFe was mixed with a nitrogen‐rich organic molecule, in this case Nicarbazin, with a mass ratio equal to 30 : 70, in Milli‐Q water (100 mL/g_SFe_) for 24 h at room temperature (RT). The dried material was pyrolyzed (SFe−P1) at 900 °C under pure N_2_ for 1 h, using a heating and cooling rate of 300 °C h^−1^. Then, the mixtures were etched in Teflon labware to remove the silica sacrificial supports by testing two aqueous solutions of different in situ HF forming agents as recently shown:^[81]^ i) NH_4_HF_2_ (10 wt %) and ii) NaF + HCl (8.5 wt % and 18 wt %, respectively). The amount of the etching solutions has been calculated according to three parameters:

– the quantity of HF formed from the dissolution of NH_4_HF_2_ and NaF in water (Equation(1–2)), that corresponds to 1 mol of HF for each mole of tested etching agent. According to Equation (3–4) and to the values of K_3_‐K_4_, this amount will mostly correspond to the moles of HF_2_^−^ in solution in the case of NH_4_HF_2_ solution, while a mixture of HF, F^−^, HF_2_^−^ and dimers of (HF)_2_ will form in the NaF solution;
– the amount of SiO_2_ in SFe−P1 measured by Thermogravimetric Analysis (TGA);
– the overall dissolution reaction of SiO_2_ by HF, which requires six moles of F^−^ for each Si mole to produce the hexafluorosilicic acid (H_2_SiF_6_) during the etching process.[^84^, ^112^]

For the tests, both a stoichiometric amount (1x) and an excess of the two agents (5x, correspondent to five times the stoichiometric amount) were used, correspondent to a molar ratio between the in situ formed HF and SiO_2_ equal to 6 and 30, respectively. In a typical procedure, the etching solutions were slowly mixed with SFe−P1 at 40 °C and kept under stirring in an oil bath for 24 h. Later on, the samples were recovered through centrifugation (9000 rpm, 30 min) and washed several times with water until the pH of the supernatant became neutral. The powders were dried at 80 °C overnight and finally pyrolyzed a second time under a slightly reducing atmosphere of N_2_/H_2_ 95/5 wt % at 900 °C for 1 h, with a heating and cooling rate of 300 °C h^−1^. The final samples were labeled as SFe−Y−X, where Y is the used HF forming agent for silica removal and X is the amount used of each agent, equal to 1 or 5, for the stoichiometric amount and the excess, respectively.

Reference Fe−N_x_−C sample for the electrochemical tests was prepared by following the same procedure herein described but using a HF/HNO_3_ mixture for the silica etching process (2 : 1 mixture of HF 25 wt % and HNO_3_ 35 wt %, RT, 3 days), according to Honig et al.^[80]^

### Electrocatalyst Characterization

*Infrared Spectroscopy* (FTIR) in the Attenuated Total Reflectance (ATR) mode was carried out using a Thermo Fisher Scientific Nicolet iS20 instrument. FTIR spectra were collected between 4000–550 cm^−1^, with a 1 cm^−1^ resolution, 32 scans. *Thermogravimetric analysis (TGA)* was performed by using a Mettler Toledo StarE system TGA/DSC1 instrument (scan range 30–1000 °C, heat rate 10 °C min^−1^, constant air flow 50 mL min^−1^). Both FTIR spectra and TGA curves were registered on bare SFe to assess the successful SiO_2_ functionalization, as well as on SFe−Y samples to verify the effective silica removal by the HF forming agents compared to SFe−P1. This was further verified by *X‐ray Fluorescence (XRF)* with a Bruker EDXRF spectrometer (Artax 200) equipped with an X‐ray tube (Mo anode) with a beam collimated down to 0.65 mm in diameter (excited sample area of 0.33 mm^2^). The working conditions were 20 kV and 1.0 mA with an acquisition time of 300 seconds.

*Transmission Electron Microscopy (TEM)* images collected at low magnification were used to confirm the absence of silica particles on SFe−Y. TEM analysis was performed with a JEOL JEM‐2100Plus TEM operating with an acceleration voltage of 200 kV, equipped with an 8‐megapixel Gatan RioTM complementary metal‐oxide‐semiconductor camera. The samples were deposited onto carbon coated Cu TEM mesh grids by drop‐casting dilute NPs dispersions in EtOH.

*Inductively Coupled Plasma‐Optical Emission spectroscopy (ICP‐OES)* was used to measure the iron amount in SFe, with an ICP‐OES Optima 7000 DV Perkin Elmer instrument, after the acid digestion in a microwave Milestone Ethos mineralizer.

The structure of SFe−Y electrocatalysts was studied with the *X‐Ray Powder Diffraction (XRPD)*, by using a Rigaku MiniFlex 600 diffractometer with 1.5406 Å Cu Kα radiation, in the 2θ range between 5–80°, 2θ step 0.02°, 1° min^−1^ scan rate. *Specific surface area (SSA)*, desorption cumulative pore volume (DCPV) and pore size distribution were measured with a Quantachrome Autosorb iQ−C instrument, where nitrogen was employed as the adsorbate at 77 K, according to BET. The samples were previously evacuated at 80 °C for 30 min, 120 °C for other 30 min and finally at 350 °C for 7 h before the analysis. The pore size distribution was calculated using the DFT method for slit pores.The surface chemical environment and composition of the materials were examined using *X‐ray photoelectron spectroscopy (XPS)* on a Kratos AXIS Supra machine with a focused Al Kɑ beam (1486.6 eV) at 15 mA and 225 W. Data was gathered from 0 to 1400 eV for survey scans, 270 to 300 eV for C 1s detailed scans, 390 to 415 eV for N 1s detailed scans, 702 to 740 eV for Fe 2p detailed scans, and 525 to 543 eV for O 1s detailed scans.

The software CasaXPS was employed for elemental analysis of the surface. A linear background was used for C 1s, N 1s, and O 1s, while a Shirley background was applied to the Fe 2p 3/2 region. Gaussian/Lorentzian peak (70 %/30 %) was chosen for peak analysis.

### Electrochemical Characterization

Linear sweep voltammetry in a three‐electrode cell (Pine WaveVortex RDE system coupled with a Pine potentiostat) was used to study the ORR electrochemical activity of the samples. The working electrode was a rotating ring disk electrode (RRDE) with the geometric area of the glassy carbon disk equal to 0.2376 cm^2^ and the platinum ring geometric area of 0.2356 cm^2^. The rotation speed of the working electrode was set to 1600 rpm. An SSG was immersed into the electrolyte solution and used as a reference electrode. The counter electrode was a titanium spring. The inks were prepared by tip sonication 5 mg of the catalyst in a solution made of 985 μL of isopropanol and 15 μL of Nafion® 5 % dispersion in water/ethanol. The obtained ink was deposited over the glassy carbon by drop casting. Before the measurements, the electrolyte was saturated with oxygen by flowing it for 20 min in the solution. In order to maintain the solution saturated with oxygen, a minimum flow of oxygen was maintained also during measurements. The potential scan range was from 1 V to 0 V versus RHE. All measured potentials were converted to potential versus RHE according to Equation 1.

(1) $$E_{RHE}=\;E_{SSG}+0.059\cdot pH+\;E_{SSG}^{0}\;$$

The potential of the ring was fixed at 1 V versus RHE. The currents generated at the disk (I_d_) and the ring (I_r_) were used to calculate the hydrogen peroxide anion produced and the number of electrons transferred (n) respectively following the Equation (2) and 3:

(2) $$\;Peroxide,\%=\;\frac{200\;\cdot \;\frac{I_{r}}{N}}{I_{d}\;+\;\frac{I_{r}}{N}}\;$$

(3) $$n=\;\frac{4I_{d}}{I_{d}+\;\frac{I_{r}}{N}}\;$$

N is the collection efficiency of the ring, it was reported by the supplier as 38 %.

## Conflict of Interests

The authors declare no conflict of interest.

1

## Supporting information

As a service to our authors and readers, this journal provides supporting information supplied by the authors. Such materials are peer reviewed and may be re‐organized for online delivery, but are not copy‐edited or typeset. Technical support issues arising from supporting information (other than missing files) should be addressed to the authors.

Supporting Information

## Data Availability

The data that support the findings of this study are available from the corresponding author upon reasonable request.

## References

[1] T. Ioroi , Z. Siroma , S. Yamazaki , K. Yasuda , Adv. Energy Mater. 2019, 9, 1801284.

[2] S. Zhang , Y. Shao , G. Yin , Y. Lin , J. Mater. Chem. A 2013, 1, 4631.

[3] L. Su , W. Jia , C. M. Li , Y. Lei , ChemSusChem 2014, 7, 361–378. 24449484 10.1002/cssc.201300823

[4] Y. He , Q. Tan , L. Lu , J. Sokolowski , G. Wu , Electrochem. Energy Rev. 2019, 2, 231–251.

[5] E. Marra , H. Grimler , G. Montserrat-Sisó , R. W. Lindström , B. Wickman , G. Lindbergh , C. Lagergren , Electrochim. Acta 2022, 435, 141376.

[6] J. Zhao , C. Fu , K. Ye , Z. Liang , F. Jiang , S. Shen , X. Zhao , L. Ma , Z. Shadike , X. Wang , J. Zhang , K. Jiang , Nat. Commun. 2022, 13, 685. 35115516 10.1038/s41467-022-28346-0 PMC8813992

[7] A. M. Gómez-Marín , J. M. Feliu , ChemSusChem 2013, 6, 1091–1100. 23640868 10.1002/cssc.201200847

[8] J. Bai , S. Ke , J. Song , K. Wang , C. Sun , J. Zhang , M. Dou , ACS Appl. Mater. Interfaces 2022, 14, 5287–5297. 35072443 10.1021/acsami.1c20823

[9] Y. Lv , H. Liu , J. Li , J. Chen , Y. Song , J. Electroanal. Chem. 2020, 870, 114172.

[10] L. Fan , H. Deng , Y. Zhang , Q. Du , D. Y. C. Leung , Y. Wang , K. Jiao , Energy Environ. Sci. 2023, 16, 1466.

[11] H. Schmies , T. Zierdt , J. Mueller-Huelstede , W. Deter , J. Lorenz , M. Wark , P. Wagner , J. Power Sources 2022, 529, 231276.

[12] H. Tang , K. Geng , D. Aili , Q. Ju , J. Pan , G. Chao , X. Yin , X. Guo , Q. Li , N. Li , Nat. Commun. 2022, 13, 7577. 36481615 10.1038/s41467-022-34489-x PMC9732346

[13] Y. Sun , S. Polani , F. Luo , S. Ott , P. Strasser , F. Dionigi , Nat. Commun. 2021, 12, 5984. 34645781 10.1038/s41467-021-25911-x PMC8514433

[14] Q. Meyer , C. Yang , Y. Cheng , C. Zhao , Electrochem. Energy Rev. 2023, 6, 16.

[15] F. Zhu , L. Luo , A. Wu , C. Wang , X. Cheng , S. Shen , C. Ke , H. Yang , J. Zhang , ACS Appl. Mater. Interfaces 2020, 12, 26076–26083. 32412233 10.1021/acsami.0c06981

[16] H. Cruz-Martínez , H. Rojas-Chávez , P. T. Matadamas-Ortiz , J. C. Ortiz-Herrera , E. López-Chávez , O. Solorza-Feria , D. I. Medina , Mater. Today Phys. 2021, 19, 100406.

[17] X. Wei , R. Z. Wang , W. Zhao , G. Chen , M. R. Chai , L. Zhang , J. Zhang , EnergyChem 2021, 3, 100061.

[18] E. Zhu , M. Wu , H. Xu , B. Peng , Z. Liu , Y. Huang , Y. Li , Adv. Funct. Mater. 2022, 32, 2203883.

[19] A. Ali , A. Laaksonen , G. Huang , S. Hussain , S. Luo , W. Chen , P. K. Shen , J. Zhu , X. Ji , Nano Res. 2024, 17, 3516–3532.

[20] C. Qin , S. Tian , W. Wang , Z. J. Jiang , Z. Jiang , Front. Chem. 2022, 10, DOI: 10.3389/fchem.2022.1073566. PMC9713252 36465867

[21] J. Cui , Q. Chen , X. Lid , S. Zhang , Green Chem. 2021, 23, 6898.

[22] E. F. Holby , G. Wang , P. Zelenay , ACS Catal. 2020, 10, 14527–14539.

[23] Y. He , G. Wu , Acc. Mater. Res. 2022, 3, 224–236.

[24] K. Kumar , L. Dubau , F. Jaouen , F. Maillard , Chem. Rev. 2023, 123, 9265–9326. 37432676 10.1021/acs.chemrev.2c00685

[25] J. R. Varcoe , P. Atanassov , D. R. Dekel , A. M. Herring , M. A. Hickner , P. A. Kohl , A. R. Kucernak , W. E. Mustain , K. Nijmeijer , K. Scott , T. Xu , L. Zhuang , Energy Environ. Sci. 2014, 7, 3135.

[26] D. R. Dekel , J. Power Sources 2018, 375, 158–169.

[27] S. Gottesfeld , D. R. Dekel , M. Page , C. Bae , Y. Yan , P. Zelenay , Y. S. Kim , J. Power Sources 2018, 375, 170–184.

[28] M. M. Hossen , M. S. Hasan , M. R. I. Sardar , J. bin Haider , Mottakin , K. Tammeveski , P. Atanassov , Appl. Catal. B: Environ. 2023, 325, 121733.

[29] X. Huang , T. Shen , T. Zhang , H. Qiu , X. Gu , Z. Ali , Y. Hou , Adv. Energy Mater. 2020, 10, 1900375.

[30] G. Wu , A. Santandreu , W. Kellogg , S. Gupta , O. Ogoke , H. Zhang , H. L. Wang , L. Dai , Nano Energy 2016, 29, 83–110.

[31] G. Wu , P. Zelenay , Acc. Chem. Res. 2013, 46, 1878–1889. 23815084 10.1021/ar400011z

[32] L. Osmieri , ChemEngineering 2019, 3, 16.

[33] A. Sarapuu , J. Lilloja , S. Akula , J. H. Zagal , S. Specchia , K. Tammeveski , ChemCatChem 2023, 15, e202300849.

[34] C. Santoro , P. Bollella , B. Erable , P. Atanassov , D. Pant , Nat. Catal. 2022, 5, 473–484.

[35] S. Gupta , S. Zhao , O. Ogoke , Y. Lin , H. Xu , G. Wu , ChemSusChem 2017, 10, 774–785. 27935237 10.1002/cssc.201601397

[36] D. Sebastiμn , A. Serov , K. Artyushkova , J. Gordon , P. Atanassov , A. S. Aricò , V. Baglio , ChemSusChem 2016, 9, 1986–1995. 27376964 10.1002/cssc.201600583

[37] M. Muhyuddin , E. Berretti , S. A. Mirshokraee , J. Orsilli , R. Lorenzi , L. Capozzoli , F. D'Acapito , E. Murphy , S. Guo , P. Atanassov , A. Lavacchi , C. Santoro , Appl. Catal. B: Environ. 2024, 343, 123515.

[38] J. Zou , C. Chen , Y. Chen , Y. Zhu , Q. Cheng , L. Zou , Z. Zou , H. Yang , ACS Catal. 2022, 12, 4517–4525.

[39] B. Jeong , D. Shin , H. Jeon , J. D. Ocon , B. S. Mun , J. Baik , H. J. Shin , J. Lee , ChemSusChem 2014, 7, 1289–1294. 24700786 10.1002/cssc.201301374

[40] N. Ramaswamy , S. Mukerjee , J. Phys. Chem. C 2011, 115, 18015–18026.

[41] H. Shen , T. Thomas , S. A. Rasaki , A. Saad , C. Hu , J. Wang , M. Yang , Electrochem. Energy Rev. 2019, 2, 252–276.

[42] X. Ge , A. Sumboja , D. Wuu , T. An , B. Li , F. W. T. Goh , T. S. A. Hor , Y. Zong , Z. Liu , ACS Catal. 2015, 5, 4643–4667.

[43] K. Artyushkova , A. Serov , S. Rojas-Carbonell , P. Atanassov , J. Phys. Chem. C 2015, 119, 25917–25928.

[44] S. A. Mirshokraee , M. Muhyuddin , J. Orsilli , E. Berretti , A. Lavacchi , C. Lo Vecchio , V. Baglio , R. Viscardi , A. Zaffora , F. Di Franco , M. Santamaria , L. Olivi , S. Pollastri , C. Santoro , Nanoscale 2024, 16, 653. 10.1039/d4nr00575a 38488880

[45] G. Zuccante , M. Acciarri , C. Lo Vecchio , I. Gatto , V. Baglio , N. Pianta , R. Ruffo , L. Navarini , C. Santoro , Electrochim. Acta 2024, 492, 144353.

[46] M. Muhyuddin , A. Friedman , F. Poli , E. Petri , H. Honig , F. Basile , A. Fasolini , R. Lorenzi , E. Berretti , M. Bellini , A. Lavacchi , L. Elbaz , C. Santoro , F. Soavi , J. Power Sources 2023, 556, 232416.

[47] S. Akula , M. Mooste , J. Kozlova , M. Käärik , A. Treshchalov , A. Kikas , V. Kisand , J. Aruväli , P. Päärn , A. Tamm , J. Leis , K. Tammeveski , J. Chem. Eng. 2023, 458, 141468.

[48] Y. Kumar , E. Kibena-Põldsepp , M. Mooste , J. Kozlova , A. Kikas , J. Aruväli , M. Käärik , V. Kisand , J. Leis , A. Tamm , S. Holdcroft , J. H. Zagal , K. Tammeveski , ChemElectroChem 2022, 9, e202200717. 10.3390/ma16145105 PMC10386096 37512381

[49] H. Wang , D. J. Liu , Curr. Opin. Electrochem. 2021, 28, 100724.

[50] F. Luo , S. Wagner , I. Onishi , S. Selve , S. Li , W. Ju , H. Wang , J. Steinberg , A. Thomas , U. I. Kramm , P. Strasser , Chem. Sci. 2021, 12, 384. 10.1039/d0sc03280h PMC8179675 34168745

[51] N. Levy , L. Elbaz , Electrocatalysis for Membrane Fuel Cells 2023, 6, 10.1002/9783527830572.ch6.

[52] A. Friedman , L. Landau , S. Gonen , Z. Gross , L. Elbaz , ACS Catal. 2018, 8, 5024–5031.

[53] L. Peles-Strahl , N. Zion , O. Lori , N. Levy , G. Bar , A. Dahan , L. Elbaz , Adv. Funct. Mater. 2021, 31, 2100163.

[54] A. Friedman , N. R. Samala , H. C. Honig , M. Tasior , D. T. Gryko , L. Elbaz , I. Grinberg , ChemSusChem 2021, 14, 1886–1892. 33629811 10.1002/cssc.202002756

[55] R. Attias , K. V. Sankar , K. Dhaka , W. Moschkowitsch , L. Elbaz , M. C. Toroker , Y. Tsur , ChemSusChem 2021, 14, 1737–1746. 33561301 10.1002/cssc.202002946

[56] W. Zhao , G. Wan , C. Peng , H. Sheng , J. Wen , H. Chen , ChemSusChem 2018, 11, 3473–3479. 30076689 10.1002/cssc.201801473

[57] Y. Luo , J. Zhang , M. Kiani , Y. Chen , J. Chen , G. Wang , S. H. Chan , R. Wang , Ind. Eng. Chem. Res. 2018, 57, 12087–12095.

[58] J. N. Liu , B. Q. Li , C. X. Zhao , J. Yu , Q. Zhang , ChemSusChem 2020, 13, 1529–1536. 31845530 10.1002/cssc.201903071

[59] S. Chen , M. Cui , Z. Yin , J. Xiong , L. Mi , Y. Li , ChemSusChem 2021, 14, 73–93. 33089643 10.1002/cssc.202002098

[60] C. Li , H. Zhang , M. Liu , F. F. Lang , J. Pang , X. H. Bu , Ind. Chem. Mater. 2023, 1, 9–38.

[61] V. A. Saveleva , K. Kumar , P. Theis , N. S. Salas , U. I. Kramm , F. Jaouen , F. Maillard , P. Glatzel , ACS Appl. Energy Mater. 2023, 6, 611–616.

[62] Y. Mun , M. J. Kim , S. A. Park , E. Lee , Y. Ye , S. Lee , Y. T. Kim , S. Kim , O. H. Kim , Y. H. Cho , Y. E. Sung , J. Lee , Appl. Catal. B: Environ. 2018, 222, 191–199.

[63] J. Shi , H. Shao , F. Yang , J. Li , L. Fan , W. Cai , J. Chem. Eng. 2022, 445, 136628.

[64] X. Xu , C. Xu , J. Liu , R. Jin , X. Luo , C. Shu , H. Chen , C. Guo , L. Xu , Y. Si , J. Alloys Compd. 2023, 939, 168782.

[65] S. H. Lee , J. Kim , D. Y. Chung , J. M. Yoo , H. S. Lee , M. J. Kim , B. S. Mun , S. G. Kwon , Y. E. Sung , T. Hyeon , J. Am. Chem. Soc. 2019, 141, 2035–2045. 30620877 10.1021/jacs.8b11129

[66] A. Serov , M. H. Robson , M. Smolnik , P. Atanassov , Electrochim. Acta 2013, 109, 433–439.

[67] A. Serov , K. Artyushkova , N. I. Andersen , S. Stariha , P. Atanassov , Electrochim. Acta 2015, 179, 154–160.

[68] L. Delafontaine , A. Cosenza , E. Murphy , Y. Liu , J. Chen , B. Sun , P. Atanassov , ACS Appl. Energy Mater. 2023, 6, 678–691.

[69] H. Adabi , A. Shakouri , N. Ul Hassan , J. R. Varcoe , B. Zulevi , A. Serov , J. R. Regalbuto , W. E. Mustain , Nat. Energy 2021, 6, 834–843.

[70] A. Serov , K. Artyushkova , P. Atanassov , Adv. Energy Mater. 2014, 4, 1301735.

[71] U. Tylus , Q. Jia , K. Strickland , N. Ramaswamy , A. Serov , P. Atanassov , S. Mukerjee , J. Phys. Chem. C 2014, 118, 8999–9008. 10.1021/jp500781v PMC4010287 24817921

[72] C. Santoro , A. Serov , R. Gokhale , S. Rojas-Carbonell , L. Stariha , J. Gordon , K. Artyushkova , P. Atanassov , Appl. Catal. B: Environ. 2017, 205, 24–33. 10.1016/j.apcatb.2016.12.013 PMC5310117 28515572

[73] A. Serov , M. H. Robson , K. Artyushkova , P. Atanassov , Appl. Catal. B: Environ. 2012, 127, 300–306.

[74] M. Mazzucato , C. Durante , Electrochim. Acta 2021, 394, 139105.

[75] S. Rojas-Carbonell , K. Artyushkova , A. Serov , C. Santoro , I. Matanovic , P. Atanassov , ACS Catal. 2018, 8, 3041–3053.

[76] Y. Huang , Y. Chen , M. Xu , T. Asset , P. Tieu , A. Gili , D. Kulkarni , V. De Andrade , F. De Carlo , H. S. Barnard , A. Doran , D. Y. Parkinson , X. Pan , P. Atanassov , I. V. Zenyuk , Mater. Today 2021, 47, 53–68.

[77] Y. Chen , Y. Huang , M. Xu , T. Asset , X. Yan , K. Artyushkova , M. Kodali , E. Murphy , A. Ly , X. Pan , I. V. Zenyuk , P. Atanassov , Mater. Today 2022, 53, 58–70.

[78] C. Lo Vecchio , A. Serov , H. Romero , A. Lubers , B. Zulevi , A. S. Aricò , V. Baglio , J. Power Sources 2019, 437, 226948.

[79] M. Malagodi , C. Canevari , L. Bonizzoni , A. Galli , F. Maspero , M. Martini , Appl. Phys. A 2013, 112, 225–234.

[80] H. C. Honig , S. Mostoni , Y. Presman , R. Z. Snitkoff-Sol , P. Valagussa , M. D'Arienzo , R. Scotti , C. Santoro , M. Muhyuddin , L. Elbaz , Nanoscale 2024, 16, 11174–11186. 38770663 10.1039/d4nr01779j

[81] A. Gentile , S. Marchionna , M. Balordi , G. Pagot , C. Ferrara , V. Di Noto , R. Ruffo , ChemElectroChem 2022, 9, e202200891.

[82] C. Streli , P. Kregsamer , P. Wobrauschek , H. Gatterbauer , P. Pianetta , S. Pahlke , L. Fabry , L. Palmetshofer , M. Schmeling , Spectrochim. Acta, Part B 1999, 54, 1433–1441.

[83] S. Mostoni , M. D'Arienzo , B. Di Credico , L. Armelao , M. Rancan , S. Dirè , E. Callone , R. Donetti , A. Susanna , R. Scotti , Ind. Eng. Chem. Res. 2021, 60, 10180–10192. 34483477 10.1021/acs.iecr.1c01580 PMC8411846

[84] S. Verhaverbeke , I. Teerlinck , C. Vinckier , G. Stevens , R. Cartuyvels , M. M. Heyns , J. Electrochem. Soc. 1994, 141, 2852.

[85] J. S. Judge , J. Electrochem. Soc. 1971, 118, 1772.

[86] P. Mctigue , T. A. Odonnell , B. Verity , Aust. J. Chem. 1985, 38, 1797–1807.

[87] H. N. Farrer , F. J. C. Rossotti , J. Inorg. Nucl. Chem. 1964, 26, 1959–1965.

[88] B. Kim , W. Lee , S. Lim , Appl. Surf. Sci. 2024, 657, 159829.

[89] A. Cosenza , L. Delafontaine , A. Ly , H. Wang , E. Murphy , Y. Liu , S. Specchia , P. Atanassov , J. Power Sources 2023, 556, 232382.

[90] M. M. Hossen , K. Artyushkova , P. Atanassov , A. Serov , J. Power Sources 2018, 375, 214–221. 10.1016/j.jpowsour.2017.11.039 PMC5738968 29398775

[91] R. Gokhale , Y. Chen , A. Serov , K. Artyushkova , P. Atanassov , Electrochem. Commun. 2016, 72, 140–143.

[92] S. Kabir , K. Artyushkova , A. Serov , B. Kiefer , P. Atanassov , Surf. Interface Anal. 2016, 48, 293–300.

[93] M. Huang , R. Ding , J. Yang , W. Shi , S. Shi , L. Chen , S. Liu , X. Yin , J. Electrochem. Soc. 2022, 169, 106507.

[94] V. C. A. Ficca , C. Santoro , E. Placidi , F. Arciprete , A. Serov , P. Atanassov , B. Mecheri , ACS Catal. 2023, 13, 2162–2175.

[95] A. Zitolo , V. Goellner , V. Armel , M. T. Sougrati , T. Mineva , L. Stievano , E. Fonda , F. Jaouen , Nature Mater. 2015, 14, 937–942. 26259106 10.1038/nmat4367

[96] J. Li , M. T. Sougrati , A. Zitolo , J. M. Ablett , I. C. Oğuz , T. Mineva , I. Matanovic , P. Atanassov , Y. Huang , I. Zenyuk , A. Di Cicco , K. Kumar , L. Dubau , F. Maillard , G. Dražić , F. Jaouen , Nature Catal. 2021, 4, 10–19.

[97] S. Kabir , K. Artyushkova , A. Serov , P. Atanassov , ACS Appl. Mater. Interfaces 2018, 10(14), 11623–11632. 29533599 10.1021/acsami.7b18651

[98] H. An , K. Min , Y. Lee , R. Na , S. E. Shim , S. H. Baeck , Mol. Catal. 2022, 530, 112589.

[99] W. Zhang , T. Cui , L. Yang , C. Zhang , M. Cai , S. Sun , Y. Yao , X. Zhuang , F. Zhang , J. Colloid. Interf. Sci. 2017, 497, 108–116. 10.1016/j.jcis.2017.02.061 28279867

[100] V. Duraisamy , K. Selvakumar , R. Krishnan , S. M. Senthil Kumar , ChemistrySelect 2019, 4, 2463–2474.

[101] H. Chen , Q. H. Li , W. Yan , Z. G. Gu , J. Zhang , Chem. Eng. J. 2020, 401, 126149.

[102] T. Wu , Y. Wang , H. Zhao , J. Dong , J. Xu , J. Colloid. Interf. Sci. 2021, 603, 706–715. 10.1016/j.jcis.2021.06.116 34225074

[103] Z. Yan , C. Dai , X. Lv , M. Zhang , X. Zhao , J. Xie , J. Alloys Compd. 2019, 773, 819–827.

[104] L. Fan , L. Zhang , X. Li , H. Mei , M. Li , Z. Liu , Z. Kang , Y. Tuo , R. Wang , D. Sun , Inorg. Chem. Front. 2022, 9, 4101–4110.

[105] M. Muhyuddin , N. Zocche , R. Lorenzi , C. Ferrara , F. Poli , F. Soavi , C. Santoro , Mater. Renew. Sustain. Energy 2022, 11, 131–141.

[106] Y. Xie , X. Yu , Z. Jin , Q. Liu , S. Liu , Y. Zhao , Z. Xiang , ASEM 2022, 1, 100006.

[107] V. Duraisamy , S. Venkateshwaran , R. Thangamuthu , S. M. Senthil Kumar , Int. J. Hydrogen Energy 2022, 47, 40327–40339.

[108] F. Sun , T. Liu , M. Huang , L. Guan , Sustain. Energ. Fuels 2023, 7, 3675–3683.

[109] J. Y. Cheon , T. Kim , Y. Choi , H. Y. Jeong , M. G. Kim , Y. J. Sa , J. Kim , Z. Lee , T. H. Yang , K. Kwon , O. Terasaki , G. G. Park , R. R. Adzic , S. H. Joo , Sci. Rep. 2013, 3, 2715. 24056308 10.1038/srep02715 PMC3779849

[110] W. Stöber , A. Fink , E. Bohn , J. Colloid. Interf. Sci. 1968, 26, 62–69.

[111] L. Mezzomo , S. Bonato , S. Mostoni , B. Di Credico , R. Scotti , M. D'Arienzo , P. Mustarelli , R. Ruffo , Electrochim. Acta 2022, 411, 140060.

[112] D. M. Knotter , J. Am. Chem. Soc. 2000, 122, 4345–4351.

