# Supplementary material for: In‐Situ HF Forming Agents for Sustainable Manufacturing of Iron‐Based Oxygen Reduction Reaction Electrocatalysis Synthesized Through Sacrificial Support Method
Source: ChemSusChem. 2024 Nov 7;18(3):e202401185. doi: 10.1002/cssc.202401185 (PMC11789974; doi:10.1002/cssc.202401185)
Supplement: Supplementary file 1 — Supporting Information [file CSSC-18-e202401185-s001.pdf]

ChemSusChem

Supporting Information

In-Situ HF Forming Agents for Sustainable Manufacturing of Iron-Based Oxygen Reduction Reaction Electrocatalysis Synthesized Through Sacrificial Support Method

Silvia Mostoni, Lorenzo Mirizzi, Alessandra Frigerio, Giovanni Zuccante, Chiara Ferrara, Mohsin Muhyuddin, Massimiliano D'Arienzo, Sara Fernanda Orsini, Roberto Scotti, Alessio Cosenza, Plamen Atanassov, and Carlo Santoro*

Supporting Information

In-situ HF forming agents for sustainable manufacturing of iron-based oxygen reduction reaction electrocatalysis synthesized through sacrificial support method

Silvia Mostoni+,[a] Lorenzo Mirizzi+,[a] Alessandra Frigerio+,[a] Giovanni Zuccante,[a,b] Chiara Ferrara,[a] Mohsin Muhyuddin,[a] Massimiliano D'Arienzo,[a] Sara Fernanda Orsini,[a] Roberto Scotti,[a] Alessio Cosenza,[c] Plamen Atanassov,[c] and Carlo Santoro [a],*

[a] Department of Materials Science, University of Milano-Bicocca U5, Via Roberto Cozzi 55, 20125, Milano (Italy)

[b] Department of Industrial Engineering, University of Padova, Via Marzolo 9, Padova, 35131, Italy

[c] Department of Chemical and Biomolecular Engineering, University of California, Irvine, CA, 92697, United States

***Corresponding author: Prof. Carlo Santoro. carlo.santoro@unimib.it**

a)

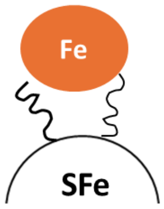

b)

EDTMS wt% (TGA)	Fe wt% (ICP-OES)	SiO_2 (wt%)
5.9 ± 0.2	1.5 wt% ± 0.1	92.6 ± 0.2

Figure S1. a) Illustrative scheme of SFe sacrificial support used for the synthesis of Fe-N-C electrocatalysts. b) Amount of EDTMS and Fe in SFe, as determined by TGA and ICP-OES analyses, respectively.[1]

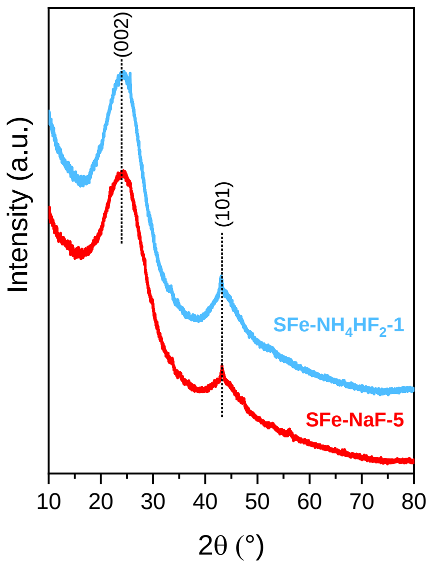

Figure S2. XRPD patterns of SFe-NH_4HF_2-1 and SFe-NaF-5.

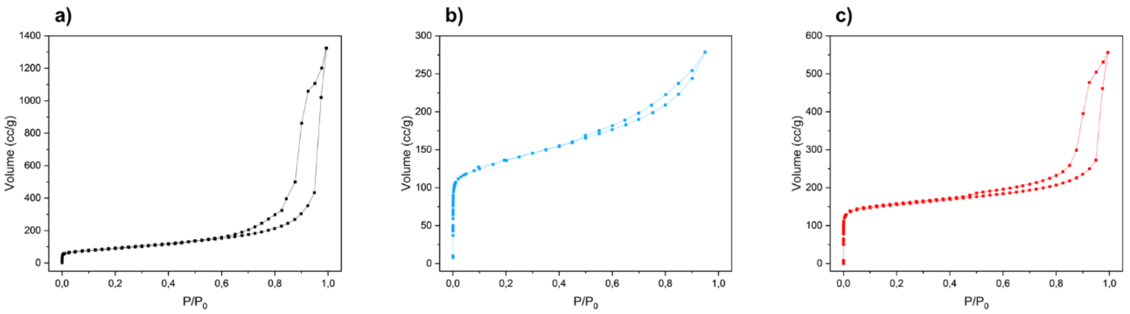

Figure S3. Nitrogen adsorption/desorption isotherms of SFe-HF (a), SFe-NH_4HF_2-1 (b) and SFe-NaF-5 (c).

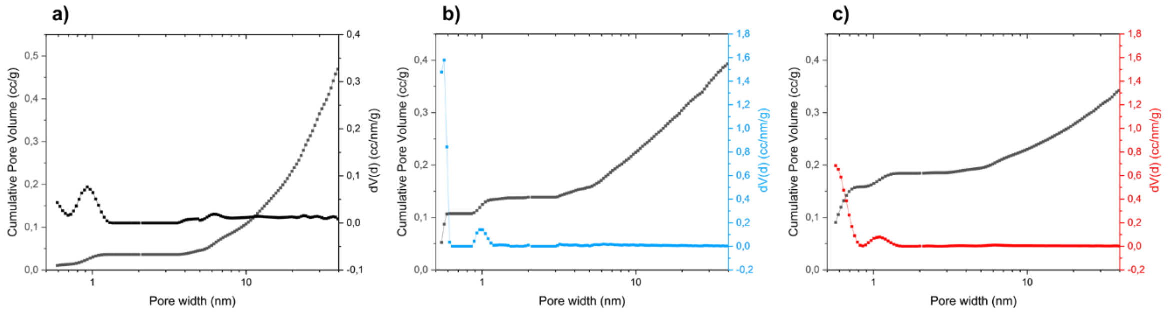

Figure S4. Cumulative pore volume and pore size distribution for SFe-HF (a), SFe-NH_4HF_2-1 (b) and SFe-NaF-5 (c).

Table S1. Micropore area and External surface area calculated by the t-Plot Method.

Sample	Micropore area (m^2 g^{-1})	External surface area (m^2 g^{-1})	SSA-BET (m^2 g^{-1})
SFe-HF	9	323	332 ± 2
SFe-NH_4HF_2-1	262	233	495 ± 2
SFe-NaF-5	419	167	586 ± 2

Table S2. Iron content estimated by ICP.

Sample	ppm	%
SFe-HF	24846.95	2.49
SFe-NH_4HF_2-1	19287.35	1.93
SFe-NaF-5	17825.65	1.78

Reference

[1] H. C. Honig, S. Mostoni, Y. Presman, R. Z. Snitkoff-Sol, P. Valagussa, M. D'Arienzo, R. Scotti, C. Santoro, M. Muhyuddin, L. Elbaz, *Nanoscale* **2024**, *16*, 11174–11186.
